# The “Moon Test”: A Step Towards Evaluating Comprehension of Educational Text through Model Mediation

**DOI:** 10.11621/pir.2021.0408

**Published:** 2021-08-03

**Authors:** Elena Vysotskaya, Anastasia Lobanova, Mariya Yanishevskaya

**Affiliations:** a Psychological Institute of Russian Academy of Education, Moscow, Russia

**Keywords:** Scientific literacy, assessment, reading comprehension, model acquisition and application, transfer from primary to secondary education

## Abstract

**Background:**

This paper addresses the issue of educational text comprehension, which is one of the major problems in secondary schools, especially when such texts are introduced in the natural sciences. Studies on text comprehension often regard reading as a standalone skill: its mechanisms are discussed from leading theoretical approaches (cognitivism, constructivism, etc.), and variables are distinguished and evaluated. Most of the researchers consider text comprehension to be active reconstruction of the meaning which the text delivers, and regard the application of the information retrieved from the text to problem-solving as the indicator for a deep comprehension level. Since we work within the framework of Cultural-Historical Activity Theory (CHAT), we consider educational text comprehension to be mediated through special content-related models which students have to acquire. Unfortunately, there are no studies which have directly linked reading, corresponding problem-solving, and working with content-related models (symbolic means, schemes); hence, with this research, we are seeking to fill in the gap.

**Objective:**

Our goal is to elaborate the perspective on educational text comprehension as mediated through mastering special modeling (symbolic) means. In this article we illustrate this approach with the “Moon test”— an assessment procedure which we designed to materialize the components of orientation of students’ action as they succeed or fail to solve problems by relying on the educational text provided.

**Design:**

We conducted the “Moon test” among the fifth graders (10-12 years old). The text, which told the students how to use the moon’s visual transformations as a calendar, was followed by 12 tasks on the topic. The tasks required using the text to master the model provided, and then solve challenging tasks which only referred to the model implicitly.

**Results:**

To analyze the results, we grouped the tasks in four blocks: 1) model acquisition; 2) mastering; 3) application; and 4) experience. The results showed a statistically significant decrease in the students’ performance on tasks of the third and the fourth blocks, which required reasonable application of the models. Further analysis of individual patterns of performance allowed us to distinguish clusters of students with different levels of success in each block.

**Conclusion:**

Our results attest to the importance of model mediation for reading comprehension and the development of scientific literacy.

## Introduction

Learning Natural Sciences in primary and secondary school demands special educational texts: they deliver the cultural templates for handling natural objects and phenomena which are to be applied in corresponding tasks. Unfortunately, as many studies show, the comprehension of these texts has always been an issue for students (NCES, 2019; Osnovnyye rezul’taty…, 2007; Zuckerman, Kovaleva, & Kuznetsova, 2013).

Issues around educational text comprehension are often considered in terms of reading competencies in general, and different groups of variables such as the texts’ characteristics and the readers’ individual skills are discussed accordingly (National Research Council, 2014). The mechanism of reading comprehension is acknowledged to be a complicated procedure aimed at reconstructing the meanings embedded in the text: active work by the students is implied (Woolley, 2011). Success may be considered the result of causal inferential processes and the application of other meta-cognitive skills (León & Escudero, 2015; McNamara, Kintsch, Songer, & Kintsch, 1996); reference to prior knowledge (Broughton, Sinatra, & Reynolds, 2010; Hynd & Alvermann, 1986; Kendeou & Van Den Broek, 2007; Mason, Gava, & Boldrin, 2008; Mikkilä-Erdmann, 2002; Wang & Andre, 1991); construction of appropriate content and textual schemas (Armbruster, 1986), and so forth. The mechanisms of text comprehension, as well as ways to improve educational texts’ design, have been investigated by many researchers from different theoretical perspectives; substantial overviews of these studies are presented elsewhere (León & Escudero, 2017; Otero & Graesser, 2014; Van Hout-Wolters & Schnotz, 2020; Woolley, 2011).

On the other hand, reading comprehension is also considered in terms of literacy— the ability to solve real-life problems using the information retrieved from texts (see PISA and PIRLS— Mullis et al., 2009; Schleicher, Zimmer, Evans, & Clements, 2009). Indeed, comprehension assessment procedures mostly include a text and corresponding tasks, which may be multiple choice questions or open tasks ( Albacete et al., 2016; León & Escudero, 2015), which are also common ways to assess disciplinary literacy. The text often concerns some meaningful problem, and a series of tasks challenge students to perform solutions based on the text (Folk, Miller, van Garderen, Lannin, & Palmer, 2020; Tamassia, & Schleicher, 2002; Zuckerman et al., 2013). In respect to the texts in textbooks, this level of comprehension (problem-solving based on the information from the text) is most desirable, since it was for this purpose that the texts were written.

There is a tendency today to consider texts somewhat “peripheral” to teaching and rather emphasize the importance and necessity of complementary activities such as solving problems (especially real-life problems), inquiry-based learning, group discussions, and so on (Khalaf, 2018; Peacock & Gates, 2000; Schilling & Hammond, 2019; National Academies…, 2017). But educational texts in general (which also include task outlines and teachers’ explanations) cannot be excluded from the learning process. In this article we suggest an approach to the issue of educational text comprehension within the Cultural-Historical Activity Theory (CHAT) framework. This allows us to pose the question: What should students do as they read the text, in order to solve the problems which rely on text comprehension?

### Educational Text Comprehension and Modeling: the CHAT Perspective

Within the Cultural-Historical and Activity Theory framework, the problem of educational text comprehension should be considered in relation to the conceptual content which these texts refer to. Students’ psychological development, the evolution of their thinking as a result of school education, relies on the acquisition and mastery of “school” concepts (Vygotsky’s term is “scientific” concepts, Vygotsky, 1986) as opposed to common everyday notions.

According to Galperin (1992), concepts are acquired through special actions which gradually evolve from the materialized form towards mental action. The materialized form of action is the most important part, as it is there that the students’ orientation is extended and made tangible through special symbolic means, such as graphs, schemas, diagrams, and so forth. The quality of these modeling tools and the extent to which students adopt them as their actual means of dealing with the problems, define the future development of the concepts and the overall quality they thus acquire.

Researchers (Salmina, 1981) distinguish between common “visuality” and “materialization.” There is an abundance of visual aids in textbooks meant to facilitate students’ comprehension by providing vivid illustrations for students to observe. “Materialization” refers to the students’ own actions, aimed at transformation of the object and operations with them (“transformative” action in CHAT terminology) and implies the design of special objects for these actions: the models. Davydov went a step further and elaborated the materialized form of action in particular, which he and his colleagues referred to as modeling or modeling actions (Davydov & Vardanyan, 1981; Rubtsov, 1994). Modeling, in Davydov’s words, is the reproduction of the “genetically initial, universal connection that determines the content and structure of the entire entity in the given concepts… in particular, object-related, graphic, or symbolic models that permit its properties to be studied `in pure form.’” (Davydov, 1990, p.174)

It is necessary to stress that in CHAT terminology, models differ from “models as representation” (a prototype) and “models as students’ mental constructs (epistemic artefacts)” (Armbruster, 1986; Gilbert, & Justi, 2016). They are content-related, substantial models which materialize the conceptual (cultural) way of thinking about the matter and how to handle it; for example, the bar diagrams for part-whole relations in early algebra (Elkonin, & Davydov, 1966; Polotskaia, 2017), the dots-in-box model for proportional reasoning about the buoyancy problem (Smith & Unger, 1997), the technological chart for natural sciences (Vysotskaya, Khrebtova, Lobanova, Rekhtman, & Yanishevskaya, 2018), and so forth. Teaching experiments within this approach have shown that these actions are essential for grasping the conceptual content of the matter, building one’s own solution for particular problems, and overcoming the pitfalls of visuality and routine experience (Davydov, 2008; Elkonin, & Davydov, 1966; Galperin, 1992).

**Figure 1.**
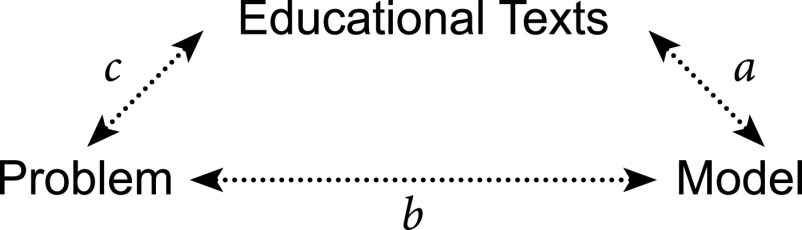
The text-model-problem triad: students’ work within each link between two “angles” (a, b, c) has to be mediated by their reference to the “third” one

*Note: Example for “a” and “c” links: What does a student do, when he understands the educational text? He reconstructs the materialized part of the action as mediated with the appropriate model provided— thus, the model actually becomes his working instrument in problem-solving. How can we tell that the student understood the text from his book? He can solve the related problems. How does this connection work? The connection works if the conceptual grounds for solving the problems were grasped from the text. How do we identify the conceptual grounds? We materialize the desired action through symbolic means, i.e., special content-related models*.

As we consider reading educational texts as part of learning in general, we have to regard text comprehension within a text-model-problem triad (*Figure 1*).

Thus, we assume that the materialized form of action and its special objects, the models, are crucial for educational text comprehension and corresponding problem-solving, as well as being the backbone of concept acquisition in general. The assessment of reading comprehension should focus on the students’ actions with models which are appropriate for the conceptual content delivered through the educational text.

### Educational Text Comprehension and Modeling: Principles of Assessment Design

The application of models to reading educational texts has received little attention, although modeling in general is considered to be important for learning the sciences as a standalone skill (Gilbert & Justi, 2016; Van Der Valk, Van Driel, & De Vos, 2007). Within the CHAT approach there are substantial studies on informational text comprehension (Zuckerman & Obukhova, 2012) and on modeling (Chudinova, 2021); yet these studies do not relate reading and modeling directly.

However, they present some principles of text comprehension assessment design which we find crucial. Critical tasks are designed to contrast “what it seems to be” and “what it is,” prompting students to follow the lead of illustrative material without applying conceptual knowledge, which eventually leads to mistakes. Galperin (Engeness, 2021; Galperin, 1992) considered such tasks to indicate the “reasonability” of the orientation content of students’ actions and related concepts. Samples of assessment tools based on the divergence between the “visual” and the “conceptual,” were designed within Galperin’s approach (Karpov, & Talysina, 1989; Pavlova, 2008; Sidneva, 2010; Sidneva, & Vysotskaya, 2019; etc.).

Following Davydov’s theory, we tried to connect students’ success in solving challenging problems to their comprehension of the corresponding educational text through their working with an appropriate model, which corresponds to the materialized form of the desired action. To bring forth the connection between educational text comprehension and appropriate model acquisition, the assessment procedure requires challenging students with tasks and problems which directly or implicitly demand using a model. Most challenging tasks should be unsolvable for those students who fail to reconstruct the general context of concept-mediated work that the text implied, and to master the materialized part of the action.

One of the feasible ways to design the tasks is to use the discrepancy between the “visual” and the “conceptual,” as mentioned above. The tasks are to be preceded by an educational text— a story which conveys the general way of solving problems concerning some matter. The materialized form of the students’ desired actions is to be provided alongside the adequate representation of the object the students are to handle (an appropriate model); we do not expect students to “invent” it themselves. While reading the text, students have to find direct guidelines and hidden clues in order to reconstruct the orientative content of actions behind the concepts. Accomplishment of the tasks, thus, would attest to the fact that the students have 1) managed to master the model presented by the text, and 2) applied the model adequately.

### The “Moon Test”: the Assessment of Students’ Work within the Text-Model-Problem Triad

To assess model-mediated reading and problem-solving among graduates of primary school, we designed the “Moon test” (Yanishevskaya, Vysotskaya, & Lobanova, 2021), which includes a short text about the visual moon’s transformations and 12 tasks referring to the topic. The Moon’s transformations were chosen, because most students are familiar with the phenomenon either through the primary natural science curriculum or through casual observation. The text is not long (less than 350 words) and reads like a story about how people used the moon’s transformations in establishing their first calendar, which allowed them to count the days by weeks and months. Here is an excerpt:

The moon is constantly changing its shape: some day we see a whole circle in the sky— a “full moon;” then only a “half ” is visible, or a beautifully outlined narrow sickle appears, which over time either “gets fat,” or “grows thin” until it disappears. In Russian such a sickle is not even called the moon, but the month.

However, one-twelfth of the year is also called a month. This is no coincidence. The moon made it possible to keep track of the days, and many peoples, including the Slavs, used the “lunar calendar,” in which the week and month were “natural” measures of the days gone by.

**Figure 2.**
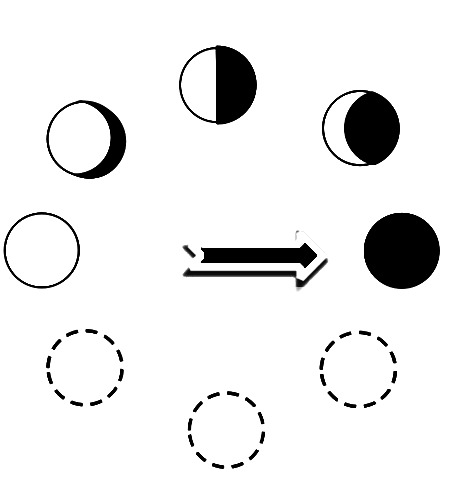
The model (the moon dial scheme) was introduced with three missing symbols. One of the tasks asked the students to complete the sequence

The subsequent 12 tasks were of four types, according to the way they engaged students in working with the model. The first three tasks (block 1) introduced the model itself— the schematic of the moon’s transformations (see the “moon dial”— *Figure 2*)— and asked the students to relate the descriptions from the text and the model (the *a* connection on *Figure 1*).

The moon dial is a symbolic representation of visible moon transformations during a one-month period; it was specially designed to scaffold the materialized form of students’ action, as they deal with measuring time passed or remaining, as people in the story did. The moon dial resembles a common clock, where weeks are counted instead of hours. This model appeals to the way people used the moon transformations they observed; thus, it is more of a model of actions than a model of the moon itself. It is not the moon as a natural object which interested people, but its surprising ability to be an accurate “timer” for people to calculate the passage of time. The central task of the “Moon test,” and the Tom Sawyer’s problem detailed below, demonstrates the necessity of this kind of representation for the calculation of time:

#9. By all accounts Tom Sawyer and Huckleberry Finn were supposed to reach the destination town in three weeks, as they floated down the river on a raft. However, after a few days, they lost count, and they could no longer say how long they had been traveling. Lights appeared on the shore. The raft moored to the shore. But is this the right place? Huck remembered clearly that they started on a night with a full moon... Throughout the cloudless night and even in the morning they looked at the sky, but the moon did not appear.

Based on this, the boys drew the necessary conclusion. Which one?

1. they had already arrived;
2. they had to raft for another week;
3. they had to raft for another two weeks;
4. they should have gone ashore a week ago.

**Figure 3.**
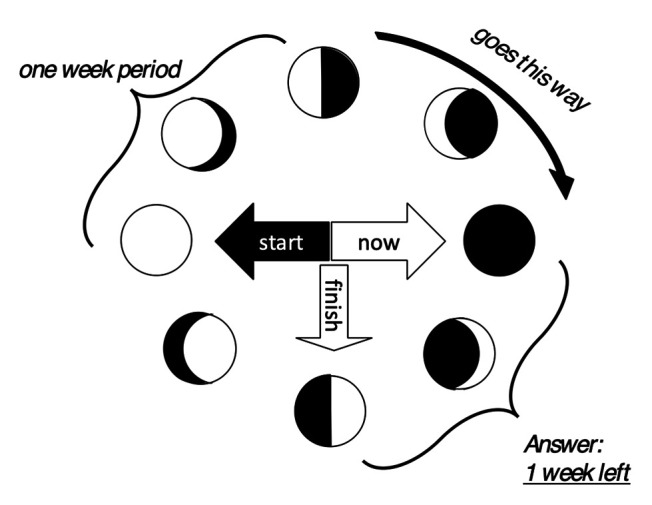
The full model design of the moon dial for TomSawyer’s task

The materialized form of the students’ action, as they found the solution for this problem, can be presented on the moon dial as follows as presented on *Figure 3*.

Solving this task requires conscious, comprehensive time calculations using the moon dial, and at the same time does not demand or even hint that the students should use the model. Moreover, the very terms of the calculation are not stated directly. If they were, the instructions would have read: “It is a new moon now. It was a full moon, when someone started on a journey that takes three weeks. Choose the correct statement.” The wording itself presents a whole text, a narration, which does not indicate the instructions for application of the model. Thus, students will have to reconstruct the whole problem on the basis of the moon dial and compose these instructions themselves.

Tom Sawyer’s task and two more tasks of the same kind comprised the third block of tasks and were the culmination of the test. They involved the *c* connection of *Figure 1*: two texts (the introductory and the task narration) were to be used to solve a real-life tricky problem— and reference to the model was the only way for students to do it. We anticipated (and the results testified to it) that these tasks would be achieved by only a minority of the students. Thus, these tasks were preceded by others that referred more clearly to the model and provided the opportunity to master the time calculation using the moon dial. They used the *b* link of the text-model-problem triad (*Fig. 1*); the students had to solve the problem posed on the moon dial, and in order to solve it, they could refer to the text. There were also tasks which involved the “moon context” but referred to other models (diagrams) which were supposed to be acquired in primary education (block 4).

Thus, the tasks which formed our diagnostics comprised four blocks: 1) matching the text and the model directly; 2) mastering the model by relying on the text; 3) referring to the model to solve difficult problems when not asked to do so explicitly; and 4) referring to models from the students’ prior experience. However, the central block of these tasks was the third one. It contained three tasks which could not be solved “directly” without applying the model implied in the text. Moreover, the necessity of using the model was not openly stated by the task, and the terms of the task did not point to the model either; thus, we considered success in these tasks as the indicator of concepts’ true functionality. The first two blocks of tasks were designed to see where the students failed when they were not able to solve the tasks of the third block, and the tasks of the fourth block were added to analyze their previous experience with using models.

Using the “Moon test” which we designed, we evaluated students’ educational text comprehension as related to their ability to reconstruct, adopt, and apply the modeling tools implied by the text.

## Methods

### Sample

We conducted the Moon test with a sample of fifth graders. The sample comprised 419 students 10–12 years old from three Moscow urban schools. The schools were not chosen for any special qualities: they were three regular state schools which agreed to participate.

### Procedure

The assessment was conducted within regular natural science lessons as individual written work in class. To exclude anxiety, the students were told that this work would not affect their grades, but at the same time they were asked to do their best. Those who did not want to participate for some reasons were not forced to; they received another assignment from their teacher. It takes about one lesson’s time to complete the test, yet many students passed their papers in earlier than that.

**Figure 4.**
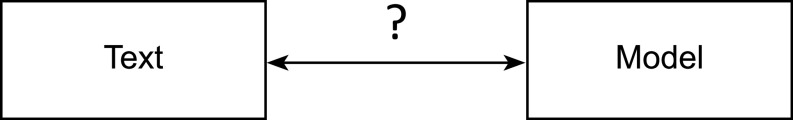
Block 1 exploited the “direct” relationship between the text and the model

Examples of the tasks from each of the four blocks are below:

Block 1: **Acquiring the model** (the acquaintance with the model was based on matching the text and the moon dial). The tasks demanded either finding information in the text to fill in the gaps in the model or vice versa (*Figure 4*).

#2. The hand on the moon dial follows the changes of moon images. How long will it take before it shows the same figure again?

#5. The moon dial drawing was not finished. Complete the drawing (*Figure 2*)

Block 2: **Mastering the model** (working directly with the model as the tasks focus on its implicit laws). The tasks required the students to explicate how the moon dial works. The “trap of visuality” was laid here on purpose. Whereas a month consists of four weeks, the moon dial has eight figures (the full moon and the new moon, two crescent moons, two almost-full moons at the opposite sides, and two half-moons). The central question went as follows:

# 6. Currently the hand is pointing at the new moon. Show the position of the hand on the moon dial after a week has passed.

One answer that “popped up” was to draw an arrow pointing at the next figure after the new moon, which would be a mistake. As was clearly stated in the text:

The “Moon Test”: A Step Towards Evaluating Comprehension of Educational Text…

…The first week the moon “grows,” and at the beginning of the second, it is already visible as a semicircle…

**Figure 5.**
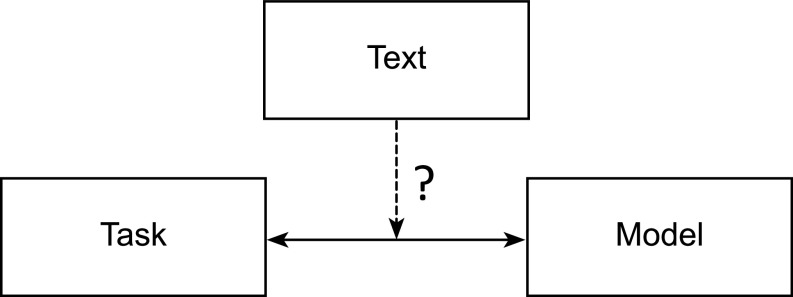
Solution of tasks from Block 2 should be mediated by referring to the text

Although the answer could be found in the text, the task demanded that the student draw an arrow on the moon dial, where one “hand” was already present. In other words, it asked him or her to transform the model according to the text, to make the “moon dial” work, to “wind up the clock.” Does the students’ comprehension of the text mediate their work over the model (*Figure 5*)?

**Figure 6.**
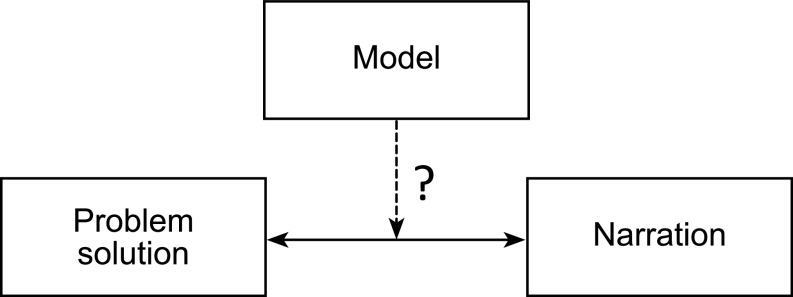
The solutions for tasks from Block 3 imply model application, though not stating it openly

Block 3: **Model application** (referring to the model initiated by the students). The tasks about Tom Sawyer and Huckleberry Finn belonged to this block: the problems introduced students to a narration and demanded an answer to a request to help the story’s characters. The necessity of referring to the model was not explicitly stated (*Figure 6*).

**Figure 7.**
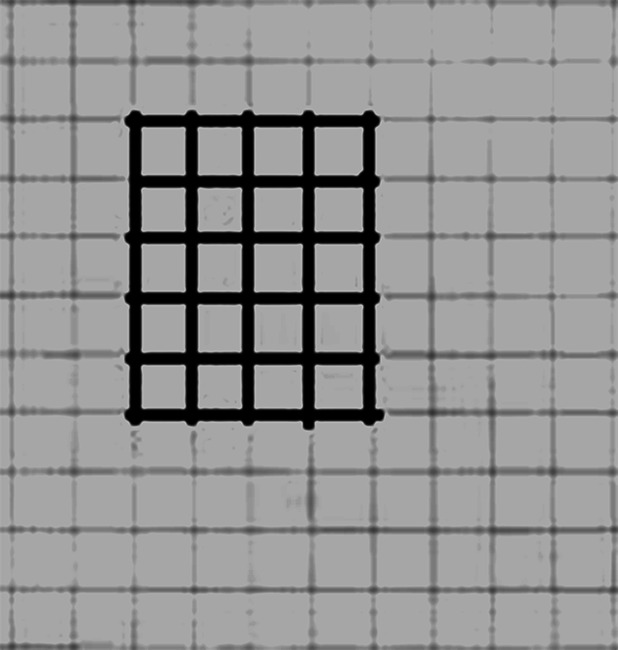
Rectangle on a grid paper, drawn to represent the mass of the Earth according to task #11

Block 4: **Model experience** (referring to other models). There were two tasks, bothof which had an essential part of the task data presented with a diagram (*Figure 7*).

# 11. The Moon weighs about 80 times less than the planet Earth. Therefore, it orbits the Earth, and not vice versa. In order for the students to clearly imagine the difference, the teacher asked them to show the masses of the Earth and the Moon on a grid paper.

One student sketched the mass of the Earth as shown in the figure. Draw how the mass of the moon should be shown in the same diagram.

Each of the task’s answers was evaluated with a 0 (the task is skipped or failed), or a 1 (the task is done correctly).

#### Data Analysis

Data Analysis was performed using Spearman’s rank correlation coefficient, the Wilcoxon signed-rank test, and Fisher’s criteria ψ.

## Results

**Figure 8.**
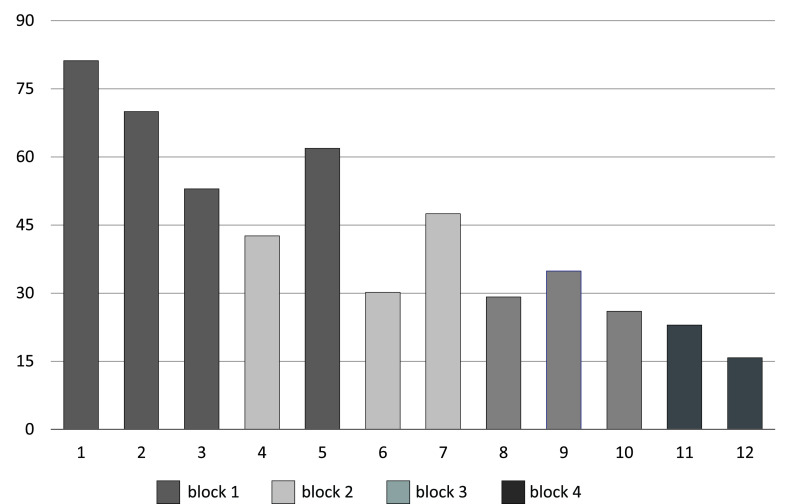
Students’ performance on the “Moon test” (percent of right answers for each task)

Below are the results for each task separately (*Figure 8*) and grouped by the four blocks (*Figure 12*).

**Figure 9.**
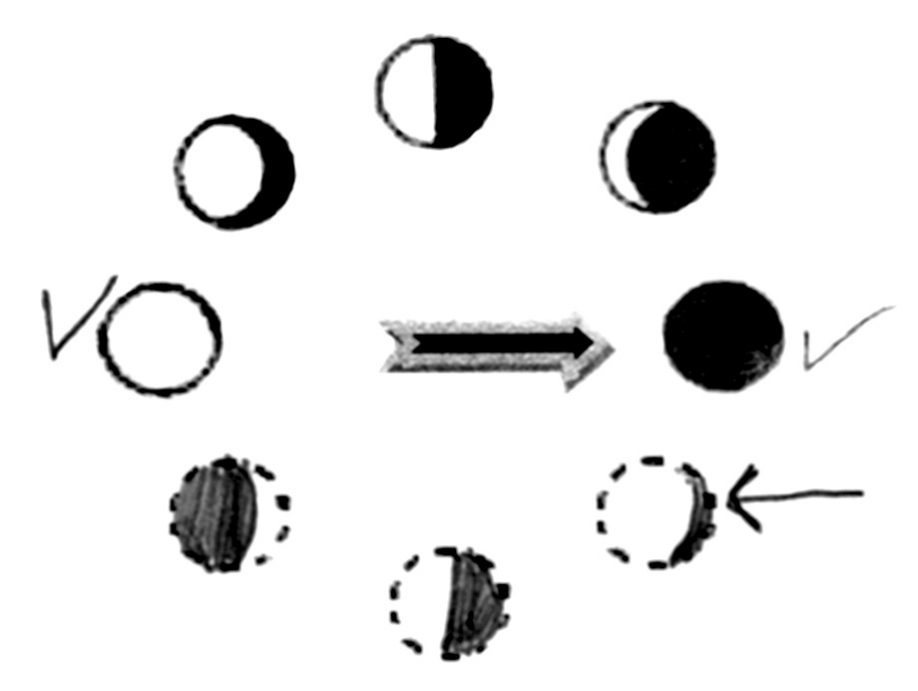
A completed moon dial

*Note: The check mark points out the full moon (task 3); the arrow points at the moon a week after the new moon (task 6), which is incorrect. The correct arrow should be pointing at the half-moon symbol*.

The qualitative analysis of the students’ answers provided us with a number of observations. The most common mistake on task 5 (“complete the moon dial,” when the three circles on the bottom are originally empty) was to color the bright part of the growing moon with the pencil, instead of coloring the dark part (see *Figure 9*). The moon, marked by the arrow, and the next symbols, would be correct if we reversed the colors.

**Figure 10.**
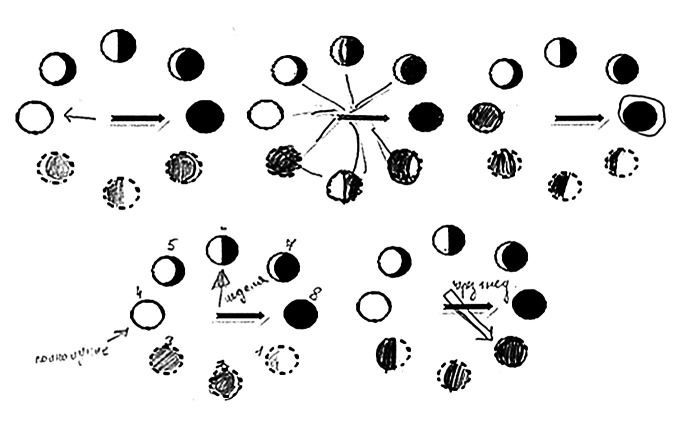
Examples of students’ mistakes on completing the moon dial and pointing the hand position after a one-week period

Task 6 was another crucial task, as it indicated whether the student “fell into the trap of visuality” and confused a week’s period with visual moon phases. As the results show (*Figure 10*), most of the students could not accomplish this task successfully. That coincided with the results of model application in the tasks that followed (the correlation between the students’ performance on task 6 and on the tasks of the third block was significant: Spearman’s rank correlation coefficient was r_s_ = 0.232, p < 0.01).

**Figure 11.**
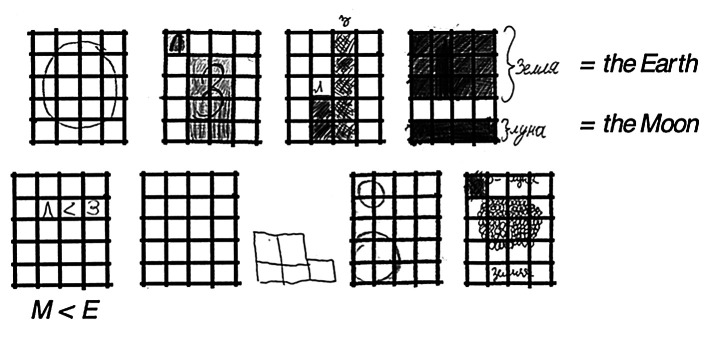
Examples of students’ mistakes on task 11

Task 11 was the one which referred to the multiplicative context while constructing diagrams. The mistakes the students made showed that many of them had no experience in modeling magnitudes and their comparison whatsoever. The task exploited the conflict between the number of visual squares— 20— and the stated difference in weight between Earth and the Moon, which is 80 times. However, many students failed to even approach the drawn rectangle as part of a symbolic representation for proportionalities between the two magnitudes (see *Figure 11* above). Students wrote or drew in the squares which represented the mass of the Earth, sketched the Moon and Earth there, and colored some of the grids to show both objects at the same time (but depicted the wrong ratio).

**Figure 12.**
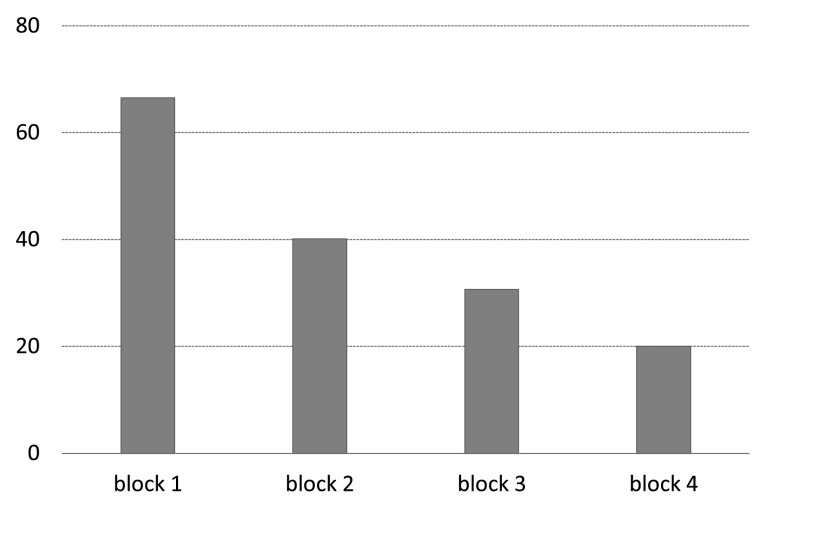
Students’ performance grouped according to the role of the model in problem-solving

*Figure 12* presents the students’ results in solving the problems grouped by the blocks outlined above. We tested the consistency of tasks for each block (Kronbach’s α was 0.688; 0.782; 0.723; 0.708 from the first to the fourth respectively). The differences between the results in each block were statistically significant (Wilcoxon signed-rank test, p < 0.01).

However, there were different patterns of task accomplishment among the students. The substantial decrease of success on the tasks of the fourth block, which is observed in the general results, was not representative for each individual’s work. The results in model acquisition (block 1) and model mastering (block 2) significantly correlated with model application (block 3); students who were careful in the tasks of first two blocks and used the model when directly told to, were more likely to succeed in difficult tasks when they referred to the model.

This distinction between the four blocks of tasks proved to be crucial for diagnostic purposes. The differences between the students’ performance on these tasks indicated the “formality” of the students’ attitude toward models: they often regarded them as mere illustrations attached to the text and did not work over them properly. That resulted in their poor problem-solving. In part, our grouping of tasks according to the role of model in problem solution can be justified by comparing the students’ results on tasks 2 and 7 (the latter is an inversion of task 2).

# 2. The hand of the moon dial turns according to the changes in moon’s appearance. How long will it take until the hand shows the same symbol again?

# 7. How will the moon dial show that exactly a month has passed?

At first glance, 7 appears easier than 2. Yet the students performed significantly worse on task 7 (Fisher’s criteria ψ = 6.94, p < 0.01), which required direct appeal to the model, rather than searching for the information provided ready-made in the text.

**Figure 13.**
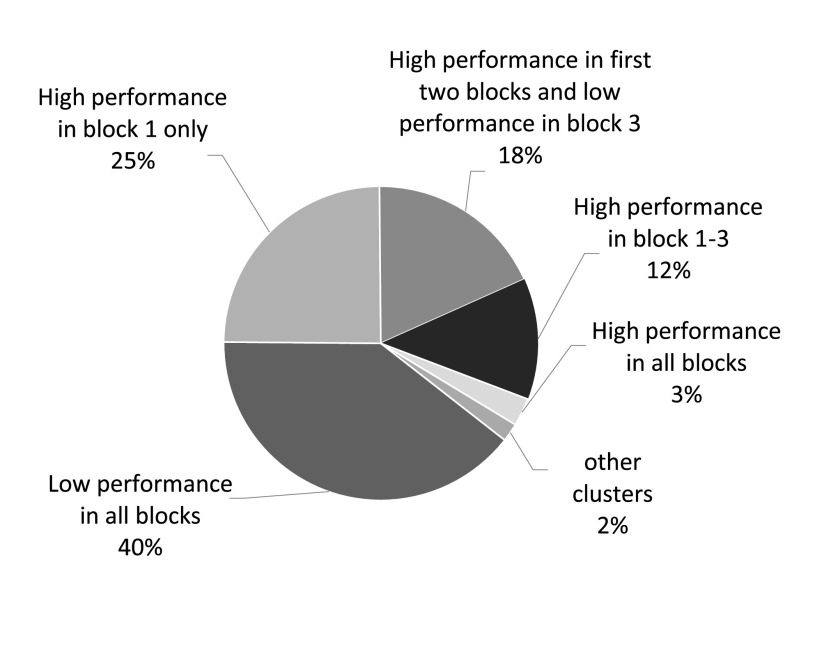
Clusters of students distinguished by patterns of their operating with model schemes

*Note: For “high” performance we considered half and more of the tasks in the block done correctly. Less than half done was considered as “low” performance*.

We have conducted a closer analysis of the most frequent patterns of students’ success within the four blocks and established six clusters of students, which to our minds represent the “state of art” in natural sciences education (see *Figure 13* above).

Many students (40%) performed poorly on all tasks, although there were only a few who skipped most of the answers. They often wrote comments that they did not understand a thing. Twenty-five percent of the students were successful on the tasks of the first block but failed the tasks of the second and third blocks. This cluster we can mark as “formal acquaintance:” these students demonstrated that they understood the drawings of the moon phases and could find appropriate pieces of information in the text to answer simple questions, but failed to transform and apply the model. Why did they fail other tasks concerning the moon dial, despite the fact that the model of the moon dial was actually helpful? Perhaps they did not consider the moon dial a working instrument. We assume that they merely thought of it as an illustration that goes alongside the text: a “broken clock” that has nothing to do with the text.

The third cluster (18%) was comprised of those who succeeded in acquiring and mastering the model, but failed to use it. These students were good at working with the dial as a special standalone object for investigation. However, dealing with the model directly did not guarantee its successful application later, although it definitely was an important step towards model mediation.

The last two clusters (high performance on all blocks [3%] and high performance on all blocks but the fourth [12%]) could be merged into one considerably larger cluster of those who performed well (15%). The difference between these two clusters may be attributed to the students’ previous education in primary school, where they might not have learned the skill of dealing with magnitudes and thus could not rely on previous skills.

## Discussion

The significant difference between the students’ performance on the tasks of the different blocks confirmed our general idea on the role of model mediation in text comprehension and problem-solving. All relationships within the text-model-problem triad are essential, as we approach the problems of science education. A direct application of the text descriptions of moon phases to solving the problems will possibly lead to right answers, but most students failed to make this link work. On the other hand, the model of the moon dial made the ideas behind time calculation, based on moon changes, tangible for students and allowed them to solve even the trickiest tasks if they used the model as a mediator for constructing a solution.

The results on tasks from blocks 3 and 4 showed that the models (moon dial and diagrams) were not functional for the majority of students. These students rather perceived them formally— as “an illustration.” A desired result of education, thus, is “de-formalization” of models: students have to apply models as a means of doing their own work. Models are symbolic representations which contain the conceptual basis for the orientation procedure in the materialized form, and thus, they are actual thought instruments for mediating solutions to problems. A discrepancy between the students’ performance on tasks of the first block, and the third and fourth blocks, is an important characteristic which has to be considered a prognostic factor towards their future learning of natural sciences (although thorough research on this matter is yet needed).

In this respect, the results, presented in the research on informational texts’ comprehension (Zuckerman, Kovaleva, & Kuznetsova, 2013; Zuckerman & Obukhova, 2012)— the absence of substantial progress during secondary education— can be regarded as an indicator of some deficits in curriculum design. As the students failed to solve problems related to the educational text, we have to question whether the content of these students’ education provided them with the substantial symbolic means to support the materialized part of their action, which is appropriate for concept-formation. Is an adequate learning situation being organized, and is the right action being demanded with challenging tasks which do not allow “bypass” (not concept-mediated) solutions?

In line with Davydov’s work, we attribute this “formalism” of modeling mainly to the content of primary school education and emphasize the necessity of implementation of Developmental Instruction principles to curriculum materials’ design (Davydov, 2008). The students’ performance on the tasks of the second block showed that working with models was the weak point which has to be elaborated. The situation, as presented by the “Moon test,” is common for regular school science classes: there is a text in the textbook followed by tasks which students often cannot solve. Teachers hence introduce model schemas and diagrams to facilitate the problems’ solutions.

There are also many ready-made models which are attached to scientific texts: the Solar system model, sequence of insects’ transformations, water cycle, and so on. However, students may not perceive the provided models in the way that teachers expect them to: they regard them as illustrations rather than working instruments. Only 40% of students were able to change the model so that it would provide the answer, which was still only the first step towards reasonable model application. There is yet special work to be organized in order for students to be able to accept and adopt the functionality of models (see our samples of developmental curricula for the natural sciences— Vysotskaya et al., 2018, 2020a, 2020b). If this kind of work is skipped, a mere demonstration with a model is unlikely to result in a successful problem-solving process.

## Conclusion

Since we follow the CHAT approach in psychology in general, and Davydov’s theory in particular, we consider educational text comprehension, as well as corresponding problem-solving, as part of a wholesome learning process, which has materialized form of action with special models at its core. Thus we assume that the evaluation of reading comprehension, as mediated through modeling, may both provide a comprehensive diagnostic tool for students’ difficulties with learning the sciences, and at the same time contribute to our understanding of the role of model mediation.

## Limitations

The comparison of our results with the performance of the same students on other diagnostics concerning educational (informational) text comprehension is one of our future tasks. A more in-depth analysis of the students’ results in block 4 (previous students’ experience with models) is also needed.
